# Inferring Function Using Patterns of Native Disorder in Proteins

**DOI:** 10.1371/journal.pcbi.0030162

**Published:** 2007-08-24

**Authors:** Anna Lobley, Mark B Swindells, Christine A Orengo, David T Jones

**Affiliations:** 1 Bioinformatics Unit, Department of Computer Science, University College London, London, United Kingdom; 2 Inpharmatica, London, United Kingdom; 3 Biocomputing Group, Department of Biochemistry, University College London, London, United Kingdom; Columbia University, United States of America

## Abstract

Natively unstructured regions are a common feature of eukaryotic proteomes. Between 30% and 60% of proteins are predicted to contain long stretches of disordered residues, and not only have many of these regions been confirmed experimentally, but they have also been found to be essential for protein function. In this study, we directly address the potential contribution of protein disorder in predicting protein function using standard Gene Ontology (GO) categories. Initially we analyse the occurrence of protein disorder in the human proteome and report ontology categories that are enriched in disordered proteins. Pattern analysis of the distributions of disordered regions in human sequences demonstrated that the functions of intrinsically disordered proteins are both length- and position-dependent. These dependencies were then encoded in feature vectors to quantify the contribution of disorder in human protein function prediction using Support Vector Machine classifiers. The prediction accuracies of 26 GO categories relating to signalling and molecular recognition are improved using the disorder features. The most significant improvements were observed for kinase, phosphorylation, growth factor, and helicase categories. Furthermore, we provide predicted GO term assignments using these classifiers for a set of unannotated and orphan human proteins. In this study, the importance of capturing protein disorder information and its value in function prediction is demonstrated. The GO category classifiers generated can be used to provide more reliable predictions and further insights into the behaviour of orphan and unannotated proteins.

## Author Summary

As a result of high throughput sequencing technologies, there is a growing need to provide fast and accurate computational tools to predict the function of proteins from amino acid sequence. Most methods that attempt to do this rely on transferring function annotations between closely related proteins; however, a large proportion of unannotated proteins are orphans and do not share sufficient similarity to other proteins to be annotated in this way. Methods that target the annotation of these difficult proteins are feature-based methods and utilise relationships between the physical characteristics of proteins and function to make predictions. One important characteristic of proteins that remains unexploited in these feature-based methods is native structural disorder. Disordered regions of proteins are thought to adopt little or no regular structure and have been experimentally linked with the correct functioning of many proteins. Additionally, disordered regions of proteins can be successfully predicted from amino acid sequence. To address the requirement for protein function prediction methods that target the annotation of orphan proteins and explore the use of information describing protein disorder, a machine learning method for predicting protein function from sequence has been implemented. The inclusion of disorder features significantly improves prediction accuracies for many function categories relating to molecular recognition. The practical utility of the method is also demonstrated by providing annotations for a set of orphan and unannotated human proteins.

## Introduction

One of the challenges of the post-genomic era is to predict the function of a protein given its amino acid sequence. Most automated function prediction methods rely upon identifying well-annotated sequence and structural homologues to transfer annotations to uncharacterised proteins (see [1,2] for a comprehensive review). Sequence similarity–based methods are relatively successful at annotating homologous proteins; however, they are not applicable to annotating orphan proteins or proteins whose relatives are not themselves functionally annotated. Currently, around 35% of proteins cannot be accurately annotated by homology-based transfer methods [3], highlighting the need for function prediction methods that are independent of sequence similarity.

ProtFun [4,5] is an ab initio feature based protein function prediction method that addresses the annotation of orphan proteins and is applicable to any protein whose sequence is known. The method makes use of sequence-based feature descriptors encoded from localisation, secondary structure, and post-translational modification predictions. Function category predictions were made using individual ensembles of neural networks trained to recognise feature patterns associated with particular functions. Similar approaches have been reported using structural properties and sequence information for prediction of enzyme classes [6,7]. One advantage of this type of approach is that features that are important in recognition of different function classes can be easily identified and quantified.

Over the past few years, there has been a growing awareness of the fundamental importance of disordered proteins in many biological functions and processes. Disordered regions of proteins can be predicted from amino acid sequence [8,9], allowing for rapid surveying of the occurrence of disorder in entire proteomes. The prevalence of disordered proteins in higher eukaryotes is thought to reflect the complexity of signalling and regulatory process within these organisms [10–12].

Disordered regions in proteins are defined as those which lack a stable well-defined 3-D structure in their native states [13,14]. Intrinsically disordered proteins may be either entirely disordered or partially disordered, characterised by long stretches of contiguously disordered residues. The presence of protein disorder is thought to confer dynamic flexibility to proteins, allowing transitions between different structural states [15]. This increased flexibility is advantageous to proteins that recognise multiple target molecules with high specificity and low affinity [13,15].

The functions of numerous disordered proteins have been characterised experimentally and include DNA and protein recognition, transcription and translation regulation, and targeted protein degradation [16–18]. The disordered regions of these proteins have been shown to be essential for their function, forcing a re-examination of the classical sequence–structure–function paradigm central to the field of structural biology and at the core of most automated function prediction algorithms [19,20].

The Protein Trinity hypothesis [19] states that protein function can arise from any of three states: ordered, molten globule, random coil, or from transitions between any or all of these states. Wright [17] defined a continuum of protein structures ranging from an unstructured conformational ensemble to mostly structured proteins containing only locally disordered regions. The functions of disordered proteins along the continuum are influenced by the presence and type of the unstructured regions. For example, disordered stretches can be flexible linker regions that allow movement between domains or can be sites of molecular attachment that become ordered on binding and give rise to functional specificity. In other proteins, disordered regions are associated with sites of post-translational modification that regulate protein–target interactions. It is clear that protein disorder is an important determinant of some protein functions; however, the value of this information remains unquantified and unexploited in current protein function prediction methods. To investigate the correlation of disorder with function, we considered the human complement of disordered proteins as predicted by DISOPRED2 [9,21]. Based on pattern analysis between the distributions of protein disorder and different function annotations, an encoding scheme for representing the occurrence of disorder in proteins is proposed. We then assess the direct influence of protein disorder in function prediction using single class Support Vector Machines [22] (SVMs) to predict individual Gene Ontology [23] (GO) categories.

## Results/Discussion

### GO Categories Enriched in Disordered Proteins

**Figure 1:**
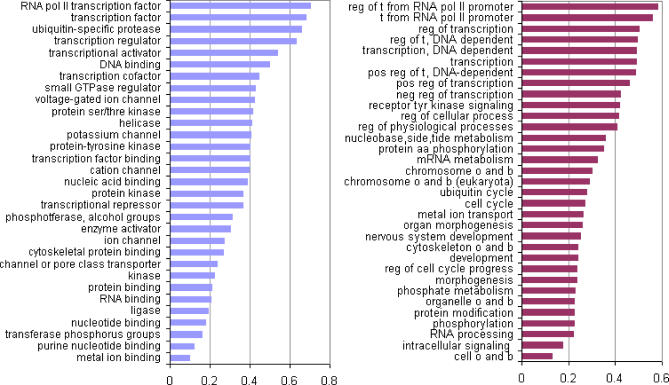
Molecular Function (A) and Biological Process (B) Categories That Are Enriched in Disordered Proteins Category names have been abbreviated: regulation (reg), transcription (t), biosynthesis (b), organisation (o), phosphorous (phos), and amino acid (aa). All reported categories are enriched in disordered proteins with *p*-value < 0.001. The *x*-axis represents the log odds ratios of observed/expected frequencies of disordered proteins in each GO category from the Fisher test. Higher odds ratios indicate greater enrichment of disordered proteins than expected by chance for the GO category.

In this analysis, a protein was considered disordered if it contained a contiguous stretch of predicted disordered residues of ≥30 amino acids. GO categories were identified that were over-represented with disordered proteins as a positive control set of categories likely to be associated with protein disorder features. 31 MF categories and 33 BP categories (Figure 1) were significantly enriched in disordered proteins at corrected *p*-values of <0.001. The cutoff was purposefully stringent to ensure virtually no false positive terms were selected.

“Transcription factor”, “DNA and protein binding”, “kinase signaling”, and “phosphorylation” molecular function (MF) categories were amongst those enriched in disordered proteins indicated by the highest log ratios of observed/expected occurrence of disordered proteins (Figure 1A). Transcription factor categories were most enriched in disordered proteins, followed by Ion channel and phosphorylation related functions. Metal-ion and nucleotide binding functions exhibited smaller yet significant enrichment in disordered proteins. “Transcription regulation”, “kinase signalling”, “RNA metabolism”, and “phosphorylation” featured in the BP categories (Figure 1B) that were enriched in disordered proteins. These categories were consistent with those functions reported both experimentally [24–26] and those reported in similar analyses of other organisms [10].

### Design of Disorder Feature Encoding Scheme

**Figure 2:**
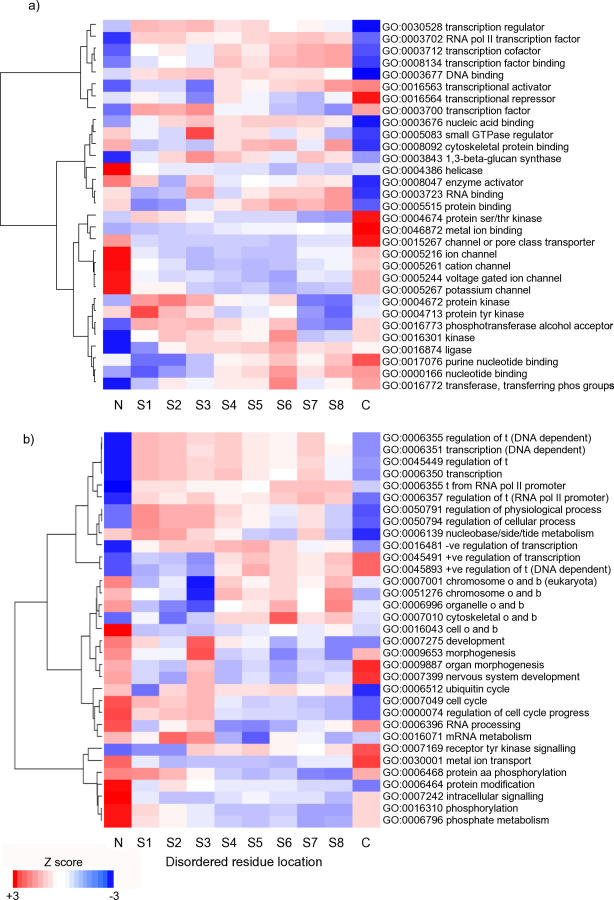
Location Features Encoding Protein Disorder for Molecular Function (A) Categories and Biological Process (B) Categories That Are Enriched in Disordered Proteins The locations are represented on the *x*-axis from N terminus through equally proportioned mid segments S1–S8 to C terminus. The clustering of GO categories was performed using Ward's hierarchical clustering method [30]. The heatmap colours reflect the significance of the association between the frequency of disordered residues within the location region and the GO category. Red blocks indicate that a high average frequency of disordered residues is associated with the GO category and the location region. Blue blocks indicate an association between low average frequency of disordered residues in the location and GO category.

We examined the distributions of protein disorder within different GO categories to ensure that the disorder features we used captured the trends and patterns relevant for function prediction. We used location descriptors to encode the position of disordered regions in proteins and length-based descriptors to distinguish short from long contiguous stretches of disordered residues. Correlations between location descriptors and GO categories were demonstrated by calculating the average frequency of disordered residues within different location windows for protein sequences annotated by a GO term (see Methods for more detail, and Figure 2). These averaged values were converted to Z-scores individually for each location window. This procedure normalised for the fact that the false positive rates for prediction of disordered residues are higher at the N and C termini of proteins than in the interior regions [10]. The Z-scores emphasized trends and sampling bias of frequencies of disordered residues directly attributable to the annotation categories. Clustering of annotation categories was performed using Ward's hierarchical method [27], which minimizes within-cluster variance measured by sums of squares error.

The location descriptors showed several trends associated with GO categories. “Transcription regulator”, “DNA binding”, and “RNA pol II Transcription factor” functions were associated with disordered residues in the protein interior, rather than at N and C termini (Figure 2A). “Transcription factor activator”, “Transcription factor repressor”, and “Transcription factor” categories showed significant associations with disordered residues toward the C terminus. Disordered residues were over-represented at the N terminus within the set of Ion Channel and more specifically potassium channel annotated proteins. A further weak association was observed between disorder at the C terminus and the ion channel categories. These observations can be confirmed by crystal structure information. For example, it has been reported that the majority of voltage-gated potassium channel proteins contain intrinsically disordered residues at their N and C terminus [28]. At the N terminus, the residues are responsible for channel inactivation [29]. The disordered residues at the C terminus are adjacent to a PDZ motif mediating binding to scaffold proteins that support the assembly of multiple ion channel subunits into a fully functioning complex [28].

Descriptors for the occurrence of different lengths of disordered regions were also constructed. The link between the length of disordered regions and sequence composition has already been described [30]. To investigate whether this observation also corresponded with functional influences, a similar clustering was performed using descriptors derived from the length distributions of disordered regions within each GO category. The region ranges were selected to reflect the shape of the entire distribution of disordered regions in the human proteome and to avoid sparse descriptors at the upper tail of the distribution (see Figure S1).

**Figure 3:**
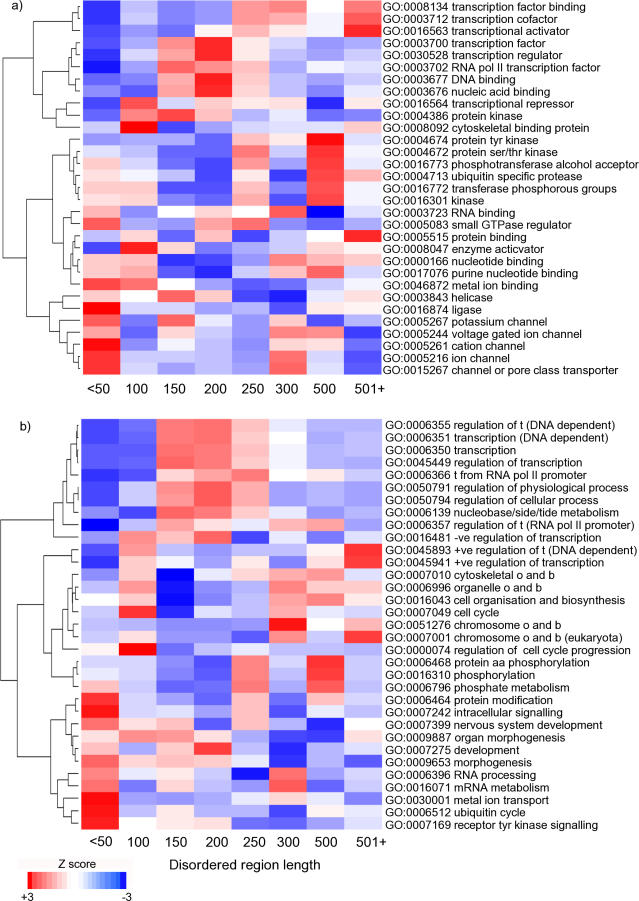
Length Dependence of Disordered Protein Functions for Molecular (A) Function Categories and Biological Process (B) Categories Enriched in Disordered Proteins The *x*-axis ranges represent ranges of disordered residue lengths; 1–50, 51–100, 101–150, 151–200, 201–250, 251–300, 301–500, and 501+. The clustering was performed using Ward's hierarchical clustering method [30]. The heatmap colours reflect the significance of the association between the frequency of disordered regions within a length range and the GO category. Red blocks indicate a significant association between high average frequency of disordered regions and GO category, and blue blocks indicate a significant association between low average frequency of disordered regions and GO category.

Clustering the GO categories by the lengths of their disordered regions (Figure 3) revealed a greater degree of function association (more significant Z-scores associated with GO categories) than for the location descriptors. Long regions of more than 500 contiguous disordered residues were over-represented in transcription-related function categories. Shorter regions (50 residues or less) were over-represented in proteins performing metal ion binding, ion channel, and GTPase regulatory functions. Proteins annotated with serine/threonine kinase and phosphatase categories were also over-represented with contiguous stretches of 300–500 disordered residue regions. Again these findings can be supported by structural evidence. Short disordered regions at the mid- to N-terminal regions in small GTPase regulatory proteins mediate a switching mechanism, enabling the protein to interact with multiple binding partners [31,32]. We demonstrate that these correlations are not simply a function of correlations between protein length and GO categories by considering “Ion Channel” and “Transcription factor binding” categories (Figure 3A). We observed a statistically significant association between shorter disordered regions and the Ion Channel GO category, yet the average length of protein within this annotation category is more than 900 amino acids. In contrast, for “Transcription factor binding”, the opposite trend is observed. The average protein length for this class is closer to 700 amino acids, and we have reported an association with long (more than 500 residue) stretches of disorder.

The correlations between function category and disorder region length may be symptomatic of the nature of the structurally disordered region. Tompa [33] described a general set of six functional classes for Intrinsically Unstructured Proteins (IUPs) that reflect their capacity to fluctuate freely in conformational space or their ability to partner molecules either permanently or transiently. It may be that the correlations displayed here between disordered region length and GO class represent the degree of structural malleability required by the protein to perform its function. For example, longer disordered regions observed in transcription regulator categories (Figure 3B) predominantly act as assemblers that are entirely unstructured and require great flexibility to function. GO categories that contain proteins whose disordered regions are predominantly display sites, for example those that are phosphorylated or involved in ubiquitination (Ubiquitin cycle in Figure 3B), require only shorter disordered regions conferring local flexibility within the protein.

The cluster groupings (Figures 2 and 3) were symptomatic of the relationships between annotation terms in the GO graph structure. Specific terms inherit annotations from general parent terms and thus share protein sequences in common. The fact that inherited terms occupied the same or similar clusters provided evidence for the robustness of the observed trends between different annotation categories. Our systematic analysis of disordered regions in the human proteome revealed significant associations between both lengths and locations of disordered regions within proteins and their different GO categories. Many of the observations can be verified by available experimental structure information, highlighting the potential value in using these attributes of disordered proteins as feature descriptors in a method to predict protein function.

### Assessing Disorder Feature Redundancy

**Figure 4:**
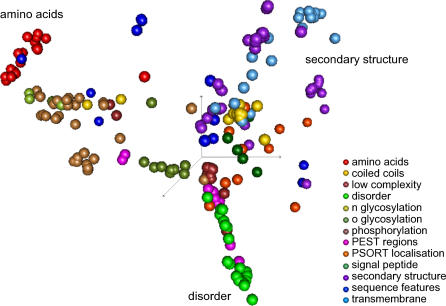
Multidimensional Scaling Plot of Feature Space Represented in Three Dimensions Feature descriptors that are closely correlated across all proteins are close together in feature space. The scale units of the plot are arbitrary and relative to the smallest correlation between feature pairs (1.27e-05) as measured by the Pearson correlation coefficient.

Including highly correlated features as inputs to machine learning algorithms often results in little increase in performance, and can sometimes result in decreased performance. To investigate relationships between the disorder features and other features to be used in function prediction, a large set of general feature descriptors was assembled (see Table S1). These were grouped into biological concepts: glycosylation or secondary structure, for example. Redundancy between feature pairs was evaluated using a feature distance matrix (1-Pearson correlation). To represent the important information in the matrix in fewer dimensions, classical Multi-Dimensional Scaling (MDS) was performed. Visualisation of the matrix using the first three dimensions as orthogonal axes (Figure 4) showed three clearly defined groupings. Amino acid composition, phosphorylation, and glycosylation features formed the first group, followed by secondary structure and transmembrane features. Disorder descriptors form a third group less extended from the origin of the plot. The shorter disorder axis reflects the fact that disordered residues are not predicted for all proteins, and, therefore, the information content within these features is comparably less than for amino acid or secondary structure features, which are generic to all proteins.

The feature relationships agreed with biological knowledge. For example, sequence features such as hydrophobicity and charge were related to the frequencies of particular amino acids within proteins. The correlations between predicted phosphorylation sites and frequency of Ser, Thr, and Tyr residues (Pearson correlation ∼0.2) were due to the fact that high frequencies of phosphorylated residues can only be observed when the relevant amino acid types occurred with a high frequency in the protein. Similarly, the frequencies of predicted O and N glycosylation sites displayed correlations with the occurrence of Asn and Ser/Thr residues. The features most closely related to disorder were random coils, PEST, and low-complexity descriptors with correlation values of 0.472, 0.211, and 0.307, respectively, at the residue frequency level. These correlations, although relatively weak, indicated that some of the information within the disorder features is also encoded by these related feature descriptors. Disordered regions in proteins frequently contain residues that are also recognised as low sequence complexity [34]; however, a region of low complexity does not always imply structural disorder. For example, fibrous proteins such as collagens and silks are rigidly structured in their native state yet contain repetitive regions of low complexity [16]. PEST motifs are degradation motifs present in proteins involved in protein phosphorylation, protein–protein interactions, and cell adhesion [35]. These motifs have been shown to be enriched in an experimentally characterised database of disordered proteins [36], and the residues that characterise the motifs represent a subset of those amino acids known to be disorder-promoting [18,37]. However, the correlations observed here between predicted occurrences of these features were small. The general spatial isolation of disorder descriptors in feature space suggested that they contain unique biological information not represented by the other features previously used in function prediction.

### Overall Influence of Disorder Features in Classification Performance

**Figure 5:**
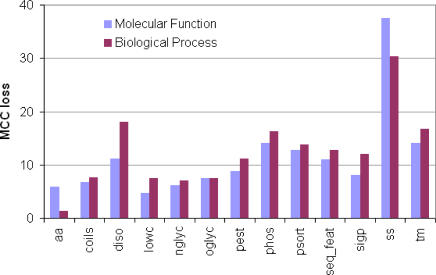
Relative Feature Importance Bar height represents median average percent loss in classifier performance for each feature group. Feature groups are abbreviated to aa (amino acid), coils (coiled coils), diso (disorder), lowc (low complexity), nglyc (n-glycosylation), oglyc (o-glycosylation), pest (PEST regions), phos (phosphorylation), psort (protein sorting), seq_feat (sequence features), sigp (signal peptide), ss (secondary structure), and tm (transmembrane regions).

Feature importance estimates for all features were collated across all GO categories using a leave-one-out elimination strategy. The histogram columns (Figure 5) represent the average percentage loss in classifier accuracy for all GO categories belonging to MF and BP ontologies, regardless of their individual category performance. Secondary structure features contributed the most to classifier performance for the majority of MF and BP categories. Disorder features were the second most important feature for BP category recognition. Amino acid composition and secondary structure contributions were higher on average for MF categories than for BPs. For all other features, the importance estimates were higher for BP categories.

Our results suggest that disorder patterns are more indicative of the biological process than the molecular activity of the protein. This is striking considering that only one-third of the proteins in the human proteome are predicted to contain significant disordered regions and the information content of the disorder feature set is comparably lower than that for secondary structure or amino acid composition. One possible reason for this observed difference lies in the respective ontology definitions. BP categories describe modules of functions that make up parts of a multi-step process [23], whereas MFs describe a protein's biochemical activity. For example, the receptor tyrosine kinase signalling BP category annotation describes the series of molecular signals generated as a consequence of a transmembrane receptor tyrosine kinase binding to its physiological ligand. Three example proteins annotated by this term are neurterin precursor a neurotrophic growth factor, Rap guanine nucleotide exchange factor, and erb-B2 receptor tyrosine-protein kinase. These proteins are all unrelated at the primary amino acid sequence and secondary structure level, yet each sequence is predicted to contain at least one 30–50 disordered residue stretch (exemplified in Figure 3B). The role of disordered regions in molecular recognition and in hub proteins in protein–protein interaction networks is well-defined [38–40]. Biologically, it would make sense that proteins that are part of the same multi-step process are more likely to co-localise and possess a common interaction surface such as a disordered region without sharing any similar sequence composition or secondary structure.

### Importance of Disorder for Individual GO Categories

To evaluate the contribution of disorder features in classification accuracy for individual categories, the performance loss was measured when disorder features were removed from each classifier using the Matthews Correlation Coefficient (MCC). This measure represents the additional value of disorder features in function prediction, accounting for both interaction and compensatory effects between features. Classifier performances were reported for 26 GO categories (Table 1) whose sensitivity at a false positive rate of 10% exceeded 50%. The significance of the improvements in correlation coefficients for individual categories were evaluated using Fisher's Z test, which considers both the magnitude of the performance increase and the strength of the correlation. The improvements that were significant at the 5% level (*p* < 0.05) were marked in bold (Table 1, column MCC+diso).

**Table 1:**
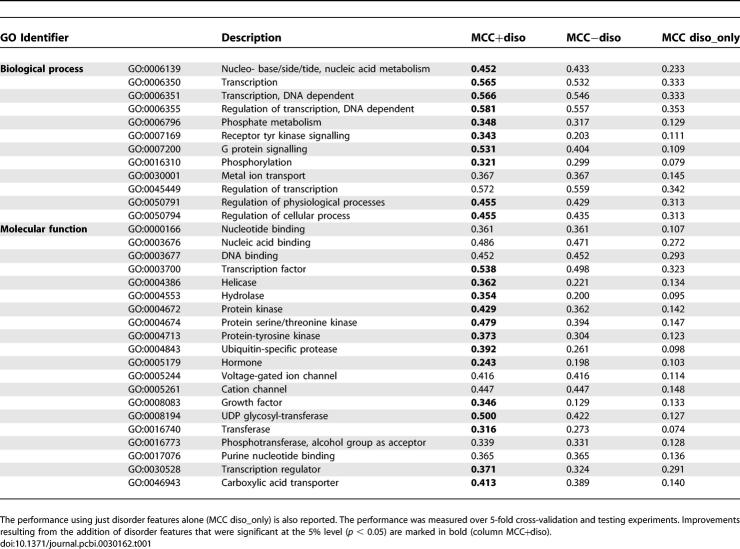
Classification Performances Measured by Matthews Correlation Coefficient (MCC) for All Features Including Disorder Features (MCC+diso) and All Features without Disorder (MCC−diso)

Eleven BP categories and 12 MF categories that were identified as enriched in disordered proteins (Figure 1) showed improvements resulting from the addition of disorder features. Several additional GO classes were identified during feature selection that required disorder features for optimal performance. Seven categories: “UDP-glycosyl transferase”, “hormone”, “growth factors”, “transferase”, “hydrolase”, and “carboxylic acid transporters” were added to the MF set of categories, and “G protein signaling” was added to the BP category set of classifiers. The most notable performance gains were observed for “protein tyrosine kinase signaling,” “G protein signaling”, “ubiquitin specific protease”, “transcription”, “protein kinase”, and “helicase” categories. For some categories (“cation-channel”, “ion channel”, “metal ion transport”, “purine-nucleotide binding”, “nucleotide binding”, and “DNA binding”), little or no performance increase resulted from the addition of disorder features. Particularly for Ion channel, Metal Ion transport, and Nucleotide binding categories, other features such as transmembrane regions or secondary structure better characterised the relationship between the primary amino acid sequence of the protein and its function.

The MCC diso–only values (Table 1) showed the correlation observed when classifiers were trained with only disorder features. Some of the BP categories relating to transcription and the Transcription factor MF category could be recognised with sensitivities of >50% at false positive rates of less than 10%, yielding Matthews correlations of ≥0.3. For these categories, the increased performance resulting from the addition of disorder features (difference between MCC+diso and MCC–diso columns in Table 1) was much lower than the correlation obtained from disorder features alone. This result can be explained by the representation of mutual information between random coil, low complexity, or PEST features reducing the magnitude of the effect of the disorder features. Conversely, for “G protein signaling” and “Receptor tyrosine kinase” BP categories and “Growth factor”, “Helicase”, “Hydrolase”, and “Ubiquitin specific protease” MF categories, the improvement resulting from the addition of disorder features was greater than the correlation obtained using disorder features alone. This finding indicates that disorder features interacted cooperatively with other features in the dataset to achieve a greater performance increase.

**Figure 6:**
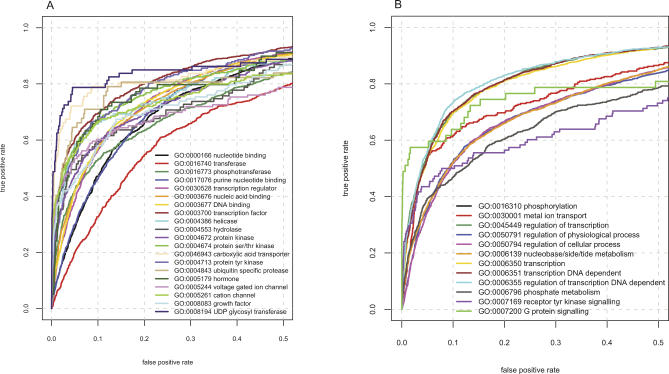
Receiver Operating Characteristics for Molecular Function (A) and Biological Process (B) Classifiers The ROC curve can be used to judge the classification sensitivities represent by the *x*-axis at different false positive rates represented on the *y*-axis.

Throughout this study, classification performance for GO categories has been reported using the MCC. This measure accounts for the imbalanced class frequencies encountered in the GO term classifiers. For completeness, the classification sensitivities obtained at 10%, 5%, and 1% false positives were reported (Table S2 and Figure 6). The number of positive class labels is also included to stress that different error rates are required for comparable performance between these classifiers. This fact is exemplified by the Receiver Operating Characteristic (ROC) curves (Figure 6 and Table S3) which vary according to class size. The curves have been zoomed in to show the sensitivities at false positive rates of below 50%. The majority of reported classifiers were capable of achieving more than 50% sensitivity at false positive rates of less than 10%. Some categories were not recognised as enriched in disordered proteins using statistical tests due to small class frequencies and low occurrences of proteins containing disordered residues. This finding highlights the advantage in using a machine learning–based approach to assess patterns of disordered features over a simple statistical approach using frequency of occurrence in recognising GO categories for which disorder is an important determinant.

In contrast to the finding that disorder features contributed more to BP category recognition, the improvements for MF and BP categories in Table 1 were slightly greater for MF than BP categories. However, these data reflect a subset of the categories for which we were able to produce accurate classifiers. This result highlights the fact that overall more BP categories utilised information from disorder features for classification than MF categories, resulting in a higher feature importance estimate overall. However, for most of these categories, we were not able to produce sufficiently accurate classifiers to be of practical use.

### Benchmarking

Our method differed from the original ProtFun method [4] in several important ways. Firstly, our predictions for structure, disorder, and transmembrane regions utilised PSI-BLAST profiles rather than single sequence predictions as feature inputs. Encoding information from sequence profiles in this manner increased the accuracy of feature predictions for those proteins that belonged to unannotated families. Second, additional secondary structure features were encoded that recorded the frequencies of helices and sheets of particular length ranges within each protein. Despite these differences, we felt it was important to provide a benchmark comparison between our method and an independent method that did not utilise disorder information. To assess the performance of the ProtFun method, the ProtFun server GO category assignments used the 14,055 annotated proteins used in this study.

**Figure 7:**
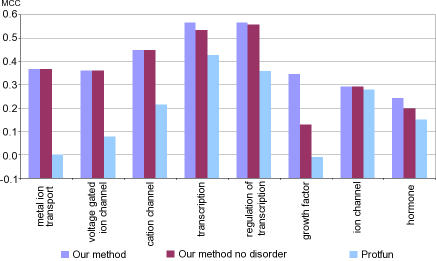
Benchmark Comparison Results Classification accuracy was assessed using Matthews correlation (*y*-axis) for eighty common GO categories for our method and for the ProtFun server. Results for our method without disorder features were shown to emphasize that performance improvements could also be the result of the use of more up-to-date training example data, feature-encoding strategies, and different machine learning algorithms.

Classifier accuracy was reported for eight common categories (Figure 7 and Table S4). The results indicated that our method outperformed the ProtFun server for all tested categories assessed using the MCC. All of these improvements were significant at the 95% level using Fisher's Z test for significance of correlation difference, except for the ion channel category. The performance of our method without disorder features (Table 1) was also reported so that the improvements in accuracy could be attributed to the use of disorder features or to the use of different training datasets and machine learning algorithms. Four of the compared function categories; “Ion Channel”, “Voltage gated ion channel”, “Cation channel”, and “Metal ion transport” did not utilise information from disorder features; therefore, improvements resulted from other methodological differences. For the remaining categories, “transcription”, “regulation of transcription”, “hormone”, and “growth factor”, the source of performance improvements were a mixture of these effects and the addition of disorder features. The greatest accuracy increase resulting directly from the addition of disorder features was observed for the “growth factor” category. For the “hormone” category, the increased accuracy resulted equally from the addition of disorder features and the algorithm and encoding differences. “Transcription” and “Regulation of transcription” accuracies were improved more by the feature encoding and more recent training datasets used than the addition of disorder features. This result was not surprising considering that the ProtFun features included low complexity, PEST regions, and random coils that overlap considerably with disorder features within these categories.

In this benchmark study, it was difficult to provide an unbiased performance measure that was comparable between the two methods. For ProtFun we were restricted to using the server output alone rather than individual neural network output scores, and any testing dataset was likely to have been used at least partially in the training of this method. However, these results indicate that our method represents a significant improvement in predicting protein function from sequence.

### Predicting GO Annotations for Unknown Proteins

The molecular recognition process and function classifiers reported have been used to classify a dataset of unannotated and orphan IPI proteins. A majority rule approach was applied to the annotations such that three of the five classifiers for each GO term must report a positive term assignment. At a confidence cutoff of 0.6 (see Figure S2 for confidence distributions), we were able to assign putative functions to 317 proteins. The majority of high confidence predictions (>0.9) were made by “transcription” and “DNA binding” MF classifiers (Table 2). Additionally, the hierarchical nature of the relationships between the GO classes can be exploited to distinguish more confident predictions. For example, many of the proteins predicted to be “regulators of transcription” also receive independent positive assignments from parent terms “transcription” and “regulation of cellular process”. The annotations have been made publicly available at http://bioinf.cs.ucl.ac.uk/anno/IPI.html.

**Table 2:**
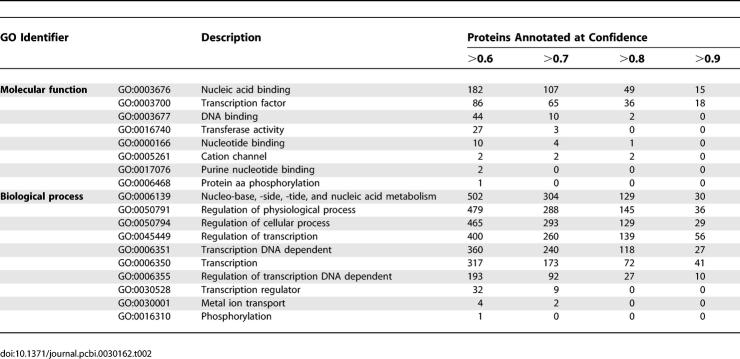
Performance of GO Term Classifiers at Different Confidence Cutoffs (Note That Classifier Power Varies with Class Size)

### Conclusions

The aim of this study was to investigate the contribution of protein disorder features in protein function prediction. This work extended numerous survey studies that report the occurrence of protein disorder within entire proteomes by identifying relevant trends and patterns of disordered regions that can be used to predict the function of proteins. Additionally, we have extended and enhanced the repertoire of GO categories that can be recognised in prediction methods by incorporating disorder features.

Disorder features contributed greater overall improvements in recognition of BP categories than MF categories. In fact, the disorder features were the second most informative feature set in BP category recognition whilst amino acid composition features were the least informative. The differences in feature importance were attributed to the differences in the descriptive nature of the two Ontologies. The anticorrelation observed between the importance of disorder features and amino acid composition for BP categories suggested that associations between disordered region length and location and BP category were not a function of similar amino acid compositions of proteins within BP categories. This finding is particularly relevant for methods that attempt to predict function or possibly protein interactions from amino acid sequence without the use of homologous sequence relationships.

The performance of 26 GO category classifiers could be improved using disorder features. Using the disorder features alone, sensitivities above 50% at false positive rates of less than 10% were obtained for some transcription-related BP categories. The results for all other categories were significantly better than random using disorder features as the sole input. These findings were impressive considering that in this study disordered residues were predicted rather than experimentally confirmed. Consequently, the estimates of feature importance were conservative and restricted by the accuracy of the disorder prediction algorithm. DISOPRED2 currently predicts 57% of residues correctly at a false positive rate of 5%. Additionally, whilst structural and compositional subtypes of disordered region have been suggested in the literature [33,41], such classifications have not yet been exploited in a method that predicts disorder from sequence. The potential value of encoding subtypes of disordered region in our function prediction method is indicated by the fact that in most cases the mutual information contained within PEST and low-complexity features was important for recognition of many of our reported GO categories.

Finally, we have demonstrated the practical application of our classifiers in predicting function for orphan and unannotated human proteins. The classifiers are applicable to any protein sequence and are well-suited to predicting putative molecular recognition functions that can then be assayed in vivo for activity, or for the purpose of target prioritisation. For the better performing classifiers, such as DNA binding and transcription related categories, identification of function from sequence can be performed. Overall, our findings reflect the importance of capturing protein disorder information and demonstrate the value of disorder features in human protein function prediction.

## Materials and Methods

### Dataset.

We used the International Protein Index (IPI) [42] as a comprehensive human protein dataset and the Gene Ontology Annotation (GOA) [43] for human. 28,057 proteins were annotated with one or more GO categories. The Cd-Hit [44,45] algorithm was used with a threshold of 60% identity to reduce overall sequence redundancy. The remaining 14,055 sequences were partitioned into five equally sized groups for cross-validation and testing. For rigorous cross-validation, the partitioning algorithm ensured that those sequences with significant homology relationships, defined as having a BLAST E-value ≤ 1e-6, were allocated to either the same training set or the same test dataset but never both. This resulted in five equally sized training and testing sets for each GO term where the maximum sequence identity between pairs of training and testing proteins did not exceed 40% sequence identity or a BLAST E-value of 10^−6^.

Positive and negative training sets for each GO term with at least 50 representative proteins were generated. Positive training examples included those proteins annotated with a particular GO term or any of its child terms in the GO hierarchy. Negative training examples included those proteins not annotated with the particular GO term or any of its children. To avoid potential class labelling errors, proteins annotated with any of the parent or less specific terms in the GO hierarchy were subsequently removed from the negative training sets. These proteins represent incomplete annotations with respect to the GO category under consideration and may belong to either the positive or negative training set for the given term.

### Over-representation analysis.

Fisher's exact test was performed under the null hypothesis that the occurrence of the GO term annotation and presence of disorder in a protein were independent. The hypothesis was rejected at *p*-values of <0.001 after applying Bonferroni multiple testing correction. The calculations were performed using the R package for statistical computing [46]. The degree of over-representation for each GO category was compared using the log odds ratio of observed over expected numbers of disordered proteins. The expected number of disordered proteins represents the background frequency, or occurrence, of disordered proteins by random chance within a sample size equivalent to the size of the GO category. This calculation yields a scale whereby values of zero indicate equality between observed and expected numbers of disordered proteins and higher values indicate a larger difference between the observed and expected values.

### Calculation of Z-scores for location and length heat maps.

For a particular GO term, the set of proteins annotated by the term or any of its child terms was considered. For location-based measures, each protein was split into ten segments; N terminus, equally proportioned segments 1 through 8, and C terminus. The frequency of disordered residues within each segment of each protein was calculated. Disordered residues were defined as those residues predicted to be disordered by DISOPRED2 at a threshold of 5% per residue false discovery rate. The set of frequencies of disordered residues within each location window for proteins annotated by each GO term was then averaged. This resulted in a set of ten average frequencies, one for each location region within each GO category. The average frequencies were Z-score normalised independently within each location window to account for the fact that the false positive rate for prediction of disordered residues is greater at the N and C termini than in the protein interior [10].

A similar approach was adopted to assess correlations between disordered region length in proteins and GO term annotations. Disordered regions in proteins were defined as contiguous stretches of ≥30 residues predicted to be disordered. The average frequency of regions that corresponded to each length range across all proteins annotated by the GO term was then calculated and converted to an independent Z-score for each length range.

### Support Vector Machines.

The Support Vector Machine [47] (SVM) is an efficient classification algorithm suitable for solving binary classification problems in high-dimensional spaces. The algorithm separates positive from negative class data by positioning a linear hyper-plane though the class examples. Often, the input data is not linearly separable, and a kernel function is required to map the data into a higher dimensional space to find the optimal separating hyperplane. The SVM was chosen over other machine learning methods of choice due to its capacity and ability to control error without causing overfitting to the data.

The *SVMlight* [48] SVM package was used to train binary classifiers for individual BP and MF GO terms using the radial basis function kernel. Kernel parameters C and γ were selected by exhaustive grid searches performed on a 272 processor Linux cluster that maximised the MCC for each classifier. MCC was chosen as a more informative measure of classifier performance than percent accuracy or error as it avoids bias resulting from unbalanced class frequencies. For example, each of the five testing sets for “GO:0045449 regulation of transcription” comprised 356 positive class examples and 1,726 negative class examples. A classification accuracy of more than 82% can be obtained by setting all predicted outcomes to be negative, whereas the MCC balances and controls for the bias in class frequencies. The MCC is similar to the Pearson correlation coefficient where 0 represents random classification and 1 implies perfect classification.

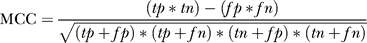



### Feature selection.

Feature selection was carried out using a recursive elimination strategy. Initially each classifier was trained and tested using all feature inputs. Optimisation of C and γ kernel parameters was performed at this stage. A single feature set was iteratively removed from the input data and the performance measured in terms of MCC. Feature attributes that did not contribute to classification performance or indeed caused improvements to performance when removed were permanently eliminated from the input data. When no further improvements were observed, a second round of parameter optimisation was performed on the final feature sets to produce final classification performance statistics. The results from feature elimination can be found in Table S2.

### Feature representation.

The features were divided into global (single values per protein) and spatial (multiple descriptors describing feature location within the protein). Global features comprised amino acid composition, sequence features, signal peptides (SignalP 3.0 [49]), and localisation information (psortII [50]). The sequence features described general protein characteristics calculated directly from the protein sequence such as molecular weight, average hydrophobicity, iso-electric point, charge, and atom counts. Local features Disorder, PEST [51] (motifs rich in proline, glutamate, serine, and threonine), coiled coils, and low-complexity residues were predicted using DISOPRED2 [52], epestfind, coils [53], and pfilt [54] algorithms with default parameter settings. Transmembrane and secondary structure content was predicted using Memsat3 [55] and PSI-Pred [56] algorithms. Post-translational modification features phosphorylation and glycosylation were predicted by NetPhos3.0, Net-N-Glyc, and Net-O-Glyc software [57]. A detailed list of descriptors for these features can be found in Table S1. All feature descriptors were scaled to between 0 and 1 before use in classification. Frequency-based descriptors such as the number of transmembrane regions were log-transformed prior to scaling.

### Disorder features.

DISOPRED2 was used to predict disordered residues for the representative protein sequence set using three iterations of PSI-BLAST [58] against the UNIPROT database release 6.0. Residues were predicted as being disordered at a false positive rate of 5%. Residue predictions were post-filtered for the presence of transmembrane regions predicted using MEMSAT 3.0 [55] set to default parameters. Predicted disordered regions were further filtered for stretches of at least 30 contiguous residues.

### Annotation of orphan and unannotated proteins.

A dataset comprising 2,157 orphan and unannotated IPI human proteins was compiled. These proteins contained one or more predicted disordered regions and represent a mixture of proteins that are either members of unannotated families or have no detectable sequence homologues by BLAST similarity searches. To calibrate comparable prediction accuracies between classifiers, the SVM outputs (distances from the separating hyperplane) were converted to posterior probabilities [59]. The probabilities were estimated from the testing datasets so that they reflect the performance of the classifiers on unannotated proteins. The predictions for the unnanotated disordered proteins have been made publicly available at http://bioinf.cs.ucl.ac.uk/anno/IPI.html.

## Supporting Information

Figure S1 Distributions of Disordered Proteins by Region Length in the Human Proteome (175 KB TIF)

Figure S2 Distribution of Scores for Orphan and Unannotated GO Terms (253 KB TIF)

Table S1 Feature Listing and Normalisation Strategy (51 KB XLS)

Table S2 Feature Selection Results for Gene Ontology Categories (18 KB XLS)

Table S3 Classifier Error Rates and GO Class Sizes (25 KB XLS)

Table S4 Benchmark Comparison Results (21 KB XLS)

## Abbreviations

BP: biological process
GO: gene ontology
IPI: International Protein Index
MCC: Matthews Correlation Coefficient
MF: molecular function
SVM: Support Vector Machine
